# 2023 Acknowledgment of *Microbiology Spectrum Ad Hoc* Reviewers

**DOI:** 10.1128/spectrum.03848-23

**Published:** 2023-12-12

**Authors:** Christina A. Cuomo

**Affiliations:** 1 Department of Molecular Microbiology and Immunology, Brown University, Providence, Rhode Island, USA

## EDITORIAL

The editors of *Microbiology Spectrum* sincerely thank all the reviewers who contributed to the peer review process during the period of November 2022 through October 2023. We are grateful for the contributions of over 4,000 reviewers who have contributed their time, effort, and expertise to the journal. We would like to specifically recognize the following *ad hoc* reviewers who have reviewed three or more articles submitted to *Microbiology Spectrum*. Your dedication to the journal and its success is commendable and greatly appreciated.

Nena A

Ghulam Abbas

Amr Abd El-Gawad

Hamad Abdelhadi

Sayed Abdelwahab

Elsayed Abdelwhab

Sahar Abduladheem

Asra'a Abdul-Jalil

Musliu Abdulkadir

Aliaa Aboulela

Abraham Terna Lan

Anastasia Accoti

La'Tonzia Adams

Kamoru Adedokun

Joshua Adkins

Amos Adler

Innocent Afeke

Muhammad Afzal

Nisheeth Agarwal

Surya Aggarwal

Christian Ahearn

Anum Ahmad

Ayaz Ahmed

Juhee Ahn

Tae-Hyuk Ahn

Tetsuya Akaishi

Hatice Akarsu

Siddiq Akbar

Muhammad Akhtar

Ramzi Alahmadi

M Alam

Laith Al-Ani

Sura Alasadi

Felipe Alberto-Lei

Alison Alencar Chaves

Hawraa Natiq AL-Fatlawy

Ali Al-Fendi

Inas Alhudiri

Intzar Ali

Yuan Allegretti

Emma Allen-Vercoe

Adriana Almeida

J Andrew Alspaugh

Hassan Al-Tameemi

John Altin

Diego Alvarez

Eva Alvarez

John Alverdy

Pedro Alves

Fairoz Al-Wrafy

Tong-Qing An

Xinwei An

Yueqing An

Lindsey Anderson

Neil Anderson

Andres Andrade Dominguez

Angel Andrade Torres

Simon Andrews

Jyot Antani

Francescopaolo Antonucci

Akira Aoki

José Argüello

Chelsie Armbruster

Alix Augusto Armero Villanueva

Gloria Arriagada

Arunangshu Aru

Asaduzzaman Asad

Victor Asensi

Usama Ashraf

Hailemariam Abrha Assress

Asaad Ataa

Joseph Ayariga

Modupe Ayilara

Hamid Badali

Taeok Bae

Jonathon Baker

Steven Baker

Nasim Balasjin

Daniel Barkan

Edwin Barrios-Villa

Pijush Basak

Sreekanth Reddy Basireddy

Anirban Basu

Denis Baurain

Clifford Beall

Kathleen Beavis

Mira Behnam

Marcel Behr

Tiago Beites

Aeriel Belk

Nabil Benazi

Kathryn Bernard

Luca Bertzbach

Sutonuka Bhar

Kanchan Bhardwaj

Disha Bhattacharjee

Somanon Bhattacharya

Souvik Bhattacharyya

Scott Biering

Fabiana Bisaro

Karishma Bisht

Basanta Biswal

Matthew Blango

Jon Blevins

Joseph Boll

Sébastien Bontemps-Gallo

Andrew Borchert

Steeve Boulant

Patricia Bradford

Leticia Bravo-Luna

Brandon Briggs

Timmie Britton

Lone Brøndsted

Jeremy Brown

Paul Brown

Laurel Burall

Laura Burrack

Jaime Bustos-Martínez

C Joaquin Caceres

Feng Cai

Yun Cai

Melissa Caimano

Gerald Call

Danielle Campbell

David Cánovas

Rafael Cantón

Meredith Carpenter

Agamemnon Carpousis

Daniel Carr

Yormaris Castillo

Maria Castrillo

Vincent Cattoir

Darina Čejková

Demet Çelebi

Miguel Cevallos

Kaan Çeylan

Ju-Hee Cha

Dipshikha Chakravortty

Martin Chan

Sittinan Chanarat

Hao-Xun Chang

Nora Chapman

Jayeshbhai Chaudhari

Gao Chen

Liang Chen

Lianmin Chen

Liu Chen

Longxin Chen

Mei-Ru Chen

Pengfei Chen

Qiusheng Chen

Yan Chen

Santenna Chenchula

Wang Cheng

Stephen Chensue

Loo Wee Chia

Rounak Chourasia

Chiranjit Chowdhury

Eng Guan Chua

Zheng Chunfu

Andrew Clark

Leanne Cleaver

Joseph Colacino

James Collins

Jennifer Colquhoun

Duan Copeland

Rebecca Corrigan

Karen Cortés Sarabia

Doug Cossar

Elena Crotti

Matthew Crotty

Luis Cruz-Vera

Jie Cui

Scott Cunningham

Ram Hari Dahal

Jan-Ulrik Dahl

Timothy Dallman

André Damasio

Matthew Deater

Boudewijn De Jonge

Rosa Del Campo

Frank DeLeo

Xianding Deng

Xin Deng

Jigar Desai

Berthony Deslouches

Marie Desnos-Ollivier

Geovane Dias-Lopes

Yohei Doi

Esteban Domingo

Caihong Dong

Tim Downing

Djamel Drider

Xiang-Dang Du

Xiangjun Du

Paul Dyson

Zoe Dyson

Mira Edgerton

Tom Edlind

Raphael Eisenhofer

Rubayet Elahi

Jeremy Ellermeier

Melinda Engevik

Hyungjin Eoh

Alice Erwin

Sandeep M Eswarappa

Oier Etxebeste

Hongjie Fan

Yanhua Fan

Matthew Faron

Na Fei

Benjamin Félix

Youjun Feng

Fernando Ferreira

Ilario Ferrocino

Paul Fey

Hannah Fischer

Elizabeth Fozo

Elaine Cristina Francisco

Simen Fredriksen

Elliot Friedman

Niels Frimodt-Møller

Xinmiao Fu

Cesira Galeotti

Sheetal Gandotra

Michael Ganzle

Erin Garcia

David Gaston

Haojie Ge

Jennifer Geddes-Mcalister

Martin Gengenbacher

Steven Gill

Erin Gloag

André Goehler

Dasantila Golemi-Kotra

Maria Gomes-Solecki

Marisa Gómez

Lan Gong

Alexandre Gouzy

David Graham

Berta Grau-Pujol

Fabio Gsaller

Bing Gu

Jingmin Gu

Rongxian Guo

Manoj Gurung

Dat Ha

Hubertus Haas

Maria Hadjifrangiskou

Ferry Hagen

Vanessa Hale

Brian Hammer

Neal Hammer

Tomoko Hanawa

Gregory Harm

Steven Harris

Travis Hartman

Akihiko Hata

Roland Hatzenpichler

Bin He

Elizabeth Hirsch

Robert Hirt

Dieneke Hoeve-Bakker

Andrew Hogan

Jeffrey Hollomon

Yaoqin Hong

Benjamin Horwitz

Saif Hossain

Lois Hoyer

Liangbin Hu

Xiaoyu Hu

Yujie Hu

Bo Huang

Shi Huang

Tingting Huang

Yao-Ting Huang

Jan Hubert

Jonathan Hulse

Evgeny Idelevich

Rustem Ilyasov

Hiroaki Inaba

İkbal Agah İnce

Kenichi Ishii

Nabila Ismail

Stanimir Ivanov

Eleanor Jameson

Francis Jenney

Jeyamalar Jeyanathan

Shoukun Ji

Cong Jiang

Yong Jiang

Maofeng Jing

Jessica Kajfasz

Girija Kaushal

Jay Keasling

Shahrbanoo Keshavarz Azizi Raftar

Sakshi Khera

Do-Kyun Kim

Todd Kitten

Kalmia Kniel

Bon-Sang Koo

Claudio Köser

Laurent Kremer

Hiroaki Kubota

Ranjeet Kumar

Anna Kuznetsova

Ling Ning Kaylie Lam

Erwin Lamping

Ke Lan

Siegmund Lang

Jeroen Langereis

Gordon Langsley

Gerald Larrouy-Maumus

Inigo Lasa

Beth Lazazzera

Shuai Le

John Lednicky

Chung-Young Lee

Sangmi Lee

Jonathan Lellouche

Jack Leo

Cheryl Leong

Kim Lewis

Bin Li

Chunhao Li

Guobang Li

Peng Li

Ruichao Li

Wentao Li

Xian-Zhi Li

Yuping Li

Yantao Liang

Fei Ling

Jian-Hua Liu

Jianzhu Liu

Pinghuang Liu

Qingyun Liu

Wenjun Liu

Xingzhong Liu

Yaqiu Liu

Małgorzata Łobocka

Shawn Lockhart

Anibal Lodeiro

Junfeng Lu

Ling Lu

Brian Luna

Antonella Lupetti

Yingfei Ma

Shruthi Magesh

Anup Majumder

Benoit Malleret

Burkhard Malorny

Melissa Martin

Michael Martin

Mathias Martins

Estibaliz Mateo

Yasufumi Matsumura

Molly Matty

Brandon Maust

John McCormick

Patrick McGann

Guruprasad Medigeshi

Stephanie Melchor

Aaron Miller

Kei Miyakawa

Masatoshi Miyakoshi

Brian Mochon

Adil Mohamed

Maria Mojica

Wendy Mok

Hector Mora-Montes

Angelica Moran

Chunlong Mu

Conrad Mullineaux

Carmen Muñoz-Almagro

Erik Munson

Bela Nagy

Wentao Ni

Robert Nicholas

Marisa Nielsen

Stefan Niemann

Elissavet Nikolaou

Guoqing Niu

Ryan Noyce

Eric Nuermberger

Dennis Nurjadi

Catherine O'Connell

Hirotaka Ode

Frank Oechslin

Kazuhiro Ogai

Dele Ogunremi

Teresa Olczak

Antonio Oliver

Laura Onyango

Daniel Ortiz

Kenji Ota

Zihao Ou

Bruno Oury

Oğulcan Özarslan

Erdal Özbek

Erkay Özgör

Samantha Palace

Robert Palmer

Fabio Palmieri

Dongli Pan

Catalina Pardo-Roa

Colin Parrish

Nicole Parrish

Chitra Pattabiraman

Natalia Perunova

Diana Pires

João Pires

Mauro Pistello

Jacqueline Plumbridge

Julia Port

Surendra Prajapat

Nicole Putnam

Qin Qi

Liang Qiao

Chaudhry R

Pawan Raghav

V Stalin Raj

Krithika Rajaram

Marie-Anne Rameix-Welti

Chad Rappleye

Ranju Rathour

Katarina Rebrosova

Guislaine Refrégier

Luis Rey

Ezio Ricca

Kristian Riesbeck

Leah Roberts

Juan Carlos Rodriguez

Eduardo Rodríguez-Román

Rebecca Rose

Özlem Şahan Yapıcıer

Romain Salle

Hyunkyu Sang

Kesavardhana Sannula

Kumar Saurav

Laximan Sawant

Joy Scaria

Oliver Schwengers

Kelly Seaton

Keun Seok Seo

Peter Setlow

Nazly Shafagati

William Shafer

Yongqi Shao

Allyson Shea

Cong Shen

Zhongyuan Shen

Tong Sheng

Vasudevan Sheshadri

Anton Sholukh

Lynn Silver

Steven Singer

Himanshu Sinha

Taweegrit Siripongboonsitti

David Šmajs

Kenneth Smith

Abigail Snyder

Hokyoung Son

Barbara Spellerberg

Richard Stanton

Brian Stevenson

Zachary Stromberg

Marc Swidergall

Alyce Taylor-Brown

Shankar Thangamani

Fabiano Thompson

Justin Thornton

Guo-Bao Tian

Kentaro Tohma

Hung-Ji Tsai

Katharine Uhteg

Faramarz Valafar

Norman Van Rhijn

Tony Velkov

Navin Kumar Verma

Rishi Vishwakarma

Jay Vornhagen

Irene Wagner-Dobler

Derek Walsh

Hong-Ning Wang

Miaoxiao Wang

Minggui Wang

Shiwei Wang

Tong Wang

Yue Wang

Brian Ward

Christopher Waters

Carl-Eric Wegner

Louis Weiss

Eric Wenzler

Lukas Weseslindtner

Stacy White

Ryan Williams

Kim Williamson

Thierry Wirth

Alan Wolfe

Alex Wong

Shengru Wu

Wei-Kai Wu

Guoqing Xia

Yan Xiang

Juan Xicohtencatl-Cortes

Jianping Xie

Jiatao Xie

Zhengde Xie

Sihong Xu

Abbas Yadegar

Guanhua Yang

Liang Yang

Yuyi Yang

Yu-Feng Yao

Fatemeh Yazdian

Wei Ye

Sheng-Hua Ying

Choo Yee Yu

Fangyou Yu

Yunsong Yu

Hassan Zafar

Irina Zakharova

Raffaele Zarrilli

Melissa Zastrow

Merve Zeden

Yuan Zeng

Chiyu Zhang

Jiachao Zhang

La-Mei Zhang

Yibei Zhang

Yanlin Zhao

Yong Zhao

Chunfu Zheng

Hao Zheng

Qingfei Zheng

Wenhui Zheng

Jin Zhong

Haijian Zhou

Jin Zhou

Bin Zhu

Kui Zhu

Lifeng Zhu

Pinkuan Zhu

Chao Zhuo

Nikola Zlatkov

Aldert Zomer

Lifang Zou

